# Influence of the Gold Nanoparticle Size on the Colorimetric Detection of Histamine

**DOI:** 10.1021/acsomega.4c02023

**Published:** 2024-07-22

**Authors:** Rolen Brian P. Rivera, Romnick B. Unabia, Renzo Luis D. Reazo, Melbagrace A. Lapening, Ryan M. Lumod, Archie G. Ruda, Jahor L. Omping, Miceh Rose D. Magdadaro, Noel Lito B. Sayson, Felmer S. Latayada, Rey Y. Capangpangan, Gerard G. Dumancas, Roberto M. Malaluan, Arnold A. Lubguban, Gaudencio C. Petalcorin, Arnold C. Alguno

**Affiliations:** † Research Center for Energy Efficient Materials (RCEEM), Premier Research Institute of Science and Mathematics (PRISM), Mindanao State University-Iligan Institute of Technology, 9200 Iligan City, Philippines; ‡ Department of Physics, Mindanao State University-Iligan Institute of Technology, 9200 Iligan City, Philippines; § Department of Chemistry, Caraga State University, Butuan City 8600, Philippines; ∥ Department of Physical Sciences and Mathematics, College of Marine and Allied Sciences, Mindanao State University at Naawan, Naawan 9023, Misamis Oriental, Philippines; ⊥ Department of Chemistry, Loyola Science Center, The University of Scranton, Scranton, Pennsylvania 18510, United States; # Center for Sustainable Polymers, MSU-Iligan Institute of Technology, Iligan City 9200, Philippines; ∇ Department of Mathematics and Statistics, Mindanao State University-Iligan Institute of Technology, 9200 Iligan City, Philippines

## Abstract

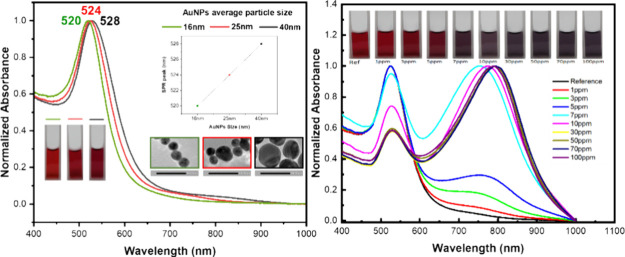

Histamine is a well-known biogenic amine (BA) that is often associated with allergic reactions and is a significant cause of foodborne illnesses resulting from the consumption of spoiled food. Detecting histamine is essential for maintaining food safety standards and preserving the quality. In this work, we developed a simple, low-cost, and rapid colorimetric method for detecting histamine. Gold nanoparticles (AuNPs) of different sizes (16, 25, and 40 nm) were synthesized by using the citrate reduction method. The particle size was controlled by adjusting the precursor molar ratio (MR), with smaller ratios leading to larger particles and a red-shift in the surface plasmon resonance (SPR) peak (520, 524, and 528 nm). The nanoparticles were allowed to interact with increasing concentrations of histamine (ranging from 1 to 100 ppm), and the changes in the absorbance spectra and color of the solution were monitored. AuNP aggregation was induced by interaction with histamine through amino and imidazole groups that will coordinate with the AuNP’s surface via electrostatic and hydrogen-bonding interactions, causing the solution to turn blue from red. The size variations of AuNPs significantly affected the colorimetric response to histamine. Among the varied sizes, 25 nm AuNPs exhibited the lowest detection limit of 0.72 μM and a linear detection range of 1–10 ppm. Notably, this sensor offered rapid detection (under 1 min) and a remarkable selectivity toward histamine analyte, highlighting its potential for practical applications.

## Introduction

Preserving food freshness is of paramount concern for both consumers and the food industry, as improper storage practices can foster the growth of microorganisms and bacteria, resulting in food spoilage through the decarboxylation of free amino acids via bacterial enzymes, leading to the production of biogenic amines (BAs) such as putrescine, cadaverine, spermine, spermidine, tyramine, phenylethylamine, histamine, and tryptamine.^1,2^ Among these, histamine is a well-known BA associated with allergic inflammatory reactions and a significant contributor to foodborne illness outbreaks caused by consuming spoiled food.^3^ High levels of histamine in foods like fish and meat can cause adverse health effects, including scombroid food poisoning.^3,4^ Improper food handling or processing can lead to excessive histamine intake, resulting in toxicological effects like vomiting and foodborne illnesses.^5^ Furthermore, histamine levels in food increase significantly during improper processing, indicating freshness and hygiene. Therefore, detecting BAs, mainly histamine, is vital for ensuring food safety and maintaining quality control.^2,6,7^

Several methodologies have been developed over time for quantifying BAs such as histamine in food, including the spectrofluorimetric method,^8^ high-performance liquid chromatography (HPLC),^9,10^ capillary electrophoresis (CE),^11,12^ gas chromatography coupled with mass spectrometry (GC–MS),^13^ thin-layer chromatography (TLC),^14^ and enzyme-linked assays.^15,16^ While these methods offer high sensitivity, they often require lengthy processing times, trained personnel, and high-quality solvents, making them expensive and time-consuming. Therefore, there is a need for a novel technique that enables rapid and sensitive detection of histamine without these drawbacks.

Colorimetric sensors, particularly those utilizing gold nanoparticles (AuNPs), offer a promising alternative for rapid and straightforward histamine detection.^17−25^ Recent studies have shown that the aggregation of gold nanoparticles (AuNPs) due to binding with histamine can produce discernible changes in color, thereby allowing for on-site analysis without the need for complex instrumentation.^23,26−28^ AuNPs interact with histamine via binding to the nitrogen atom, resulting in nanoparticle aggregation. This aggregation affects the surface plasmon resonance properties of the AuNPs, inducing color changes that can be observed and measured.

Several studies have utilized AuNPs as probes for histamine detection through induced aggregation.^23,27−29^ Previous research frequently involves the functionalization of AuNPs with specific ligands to facilitate steric hindrance and targeted analyte aggregation.^25,30^ Nevertheless, this process necessitates multiple steps, contributing to an increased production time and cost. Conversely, several literature studies have reported the utilization of unmodified AuNPs for colorimetric histamine detection.^23,26−29,31^ However, these approaches require a long incubation time during the reaction. Additionally, the influence of various sizes of AuNPs, particularly without functionalization, on their colorimetric response to histamine has scarcely been explored.

To address this gap, it is pertinent to consider the significance of the AuNPs size in colorimetric sensing. Controlling the size of nanoparticles is technologically essential due to their significant influence on the optical, electrical, and catalytic properties of AuNPs. These properties rely on surface-to-volume ratio, surface plasmon resonance, and environmental conditions.^32,33^ Hence, this study investigates the influence of the sizes of unmodified AuNPs on their colorimetric response to histamine, which is a critical marker of foodborne illness. Through the facile synthesis of AuNPs of varied sizes by employing low precursor concentrations and varying precursor molar ratios and assessing their efficacy as colorimetric sensors for histamine detection, we aim to optimize the color response of the sensor to histamine and reduce the detection time, ultimately enhancing its sensing capabilities for potential practical applications.

## Experimental Section

### Materials

Gold(III) chloride hydrate (HAuCl_4_) (99.995% trace metals basis), trisodium citrate dihydrate (ACS reagent, ≥99.0%), and histamine (Analytical Standard, 97%) were acquired from Sigma-Aldrich. Ultrapure water (18.2 mΩ, Direct-Q, Millipore SAS) was used in all procedures.

### Synthesis of Citrate-Capped AuNPs

In a standard synthesis of AuNPs, a 50 mL solution of 0.5 mM HAuCl_4_ was prepared in a flask. Simultaneously, a 34.0 mM (1.0 wt %) trisodium citrate (Na_3_Ct) solution was also prepared. The flask containing the HAuCl_4_ solution was then vigorously stirred while heated in a water bath to 95 °C. To prevent contamination and solvent evaporation during synthesis, the flask was covered with a Petri dish. Once the desired temperature was attained, a specific volume of Na_3_Ct was rapidly added to the HAuCl_4_ solution, resulting in a gradual color transition from yellow to wine-red. The MR of Na_3_Ct to HAuCl_4_ (1.5, 2.0, and 2.5) was carefully controlled as the primary parameter to achieve the targeted particle size.^34^ Synthesis was considered complete once the solution’s color ceased to change, typically requiring approximately 2, 4, and 5 min for stabilization at MR values of 1.5, 2.0, and 2.5, respectively.^35^ The solutions were allowed to cool naturally to room temperature and were subsequently stored at 4 °C for further use.

### Colorimetric Tests Were Performed with Histamine Solution

The analyte solutions were prepared by dissolving a certain amount of histamine precursor in ultrapure water with concentrations of 1–100 ppm in odd increments. A 50 μL aliquot of analyte solution was dropped in 2.0 mL of AuNPs and was allowed to react within 30 s to 1 min for colorimetric testing. The tested solution was photographed, and a chromameter was used to verify any color variation with an increasing histamine concentration. The tested solution was also characterized using UV–vis to obtain the absorbance spectra and assess the changes in the surface plasmon resonance (SPR) peak of AuNPs after adding analytes. To investigate the sensing mechanism of histamine, as-synthesized AuNPs and AuNPs with 100 ppm of histamine added were further characterized.

### Selectivity Test

Selectivity analyses were undertaken to evaluate the colorimetric responses of AuNPs toward various analytes, including organic solvents, weak acids, and diverse biological substances containing amino groups such as putrescine, cadaverine, inosine, and histamine. A 2.0 mL solution of AuNPs was introduced into a cuvette, followed by the addition of 100 μL of analyte solutions. The resulting mixture was allowed to undergo a reaction for 5 min. After this reaction period, the absorbance spectra and changes in the SPR peaks were observed using UV–visible spectroscopy. The color of the solution and the absorbance ratio of A_650_/A_520_ were compared to assess the selectivity. Higher values indicate aggregation, which changed the color from red to blue.

### Time-Dependent Stability Test

Investigating the colloidal stability of AuNPs solutions over time is crucial for their practical applications, such as colorimetric sensors for food spoilage monitoring. Stable solutions should exhibit minimal SPR peak weakening and no visible color changes during storage. To assess this stability, the time-dependent behavior of AuNP solutions stored at 4 °C for 30 days was systematically studied. We then analyzed 2 mL aliquots using UV–visible spectroscopy, focusing on changes in the SPR peak intensity and wavelength as a measure of stability.

### Measurement and Characterization

A JEM 2100 Plus LaB6 transmission electron microscope (TEM) (JEOL, Japan) characterized the particle size and morphology of AuNP solutions and their aggregation upon adding histamine. Dynamic light scattering (DLS) on a Nanotrac Wave II Analyzer (Microtrac, Inc., Pittsburgh, PA, USA) provided hydrodynamic diameter sizes, zeta potential, and polydispersity index (PDI) of the colloidal solutions before and after histamine addition. UV–vis absorption spectra of AuNPs and AuNPs in the presence of histamine were acquired at room temperature with a Thermo-Scientific GENESYS 10S spectrometer (Thermo-Scientific, Massachusetts, USA). Fourier transform infrared (FTIR) spectra were recorded at room temperature on a Shimadzu IR-TRACER100 spectrometer (Shimadzu, Japan) to identify the characteristic bonds associated with AuNPs and the presence of histamine.

## Results and Discussion

**Figure 1:**
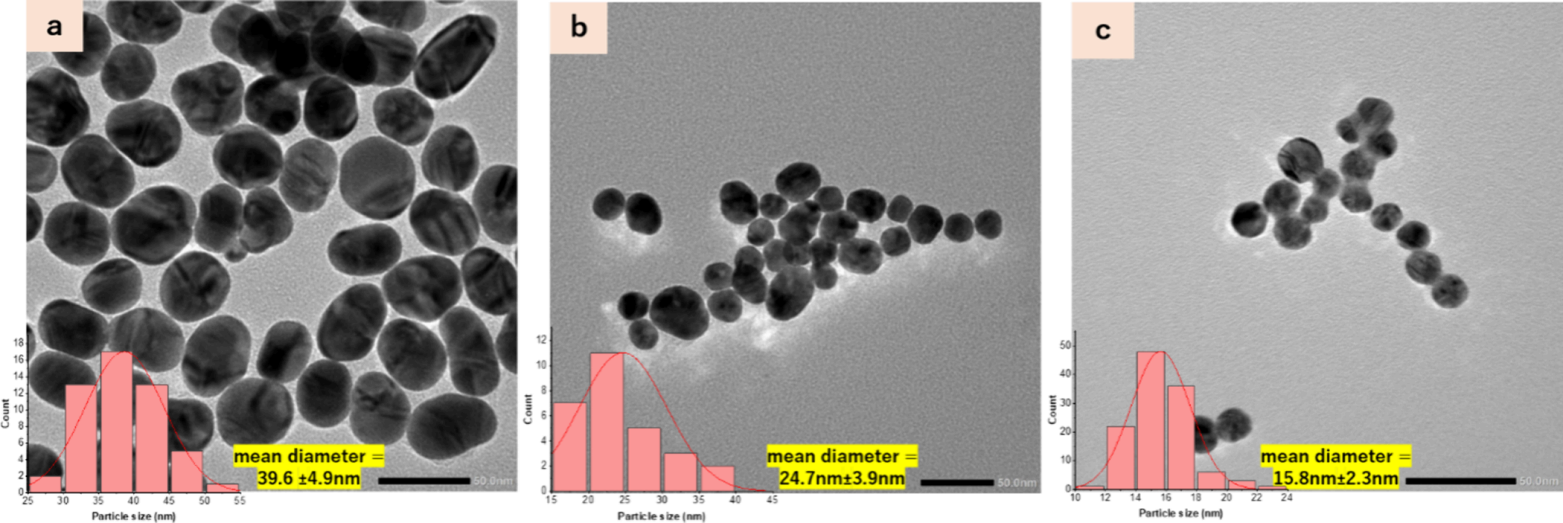
Transmission electron microscopy (TEM) images showing the particle size of gold nanoparticles with varied molar ratios of Na_3_Ct to HAuCl_4_: (a) 1.5, (b) 2.0, and (c) 2.5. The inset is a graph of its particle diameter histogram.

**Figure 2:**
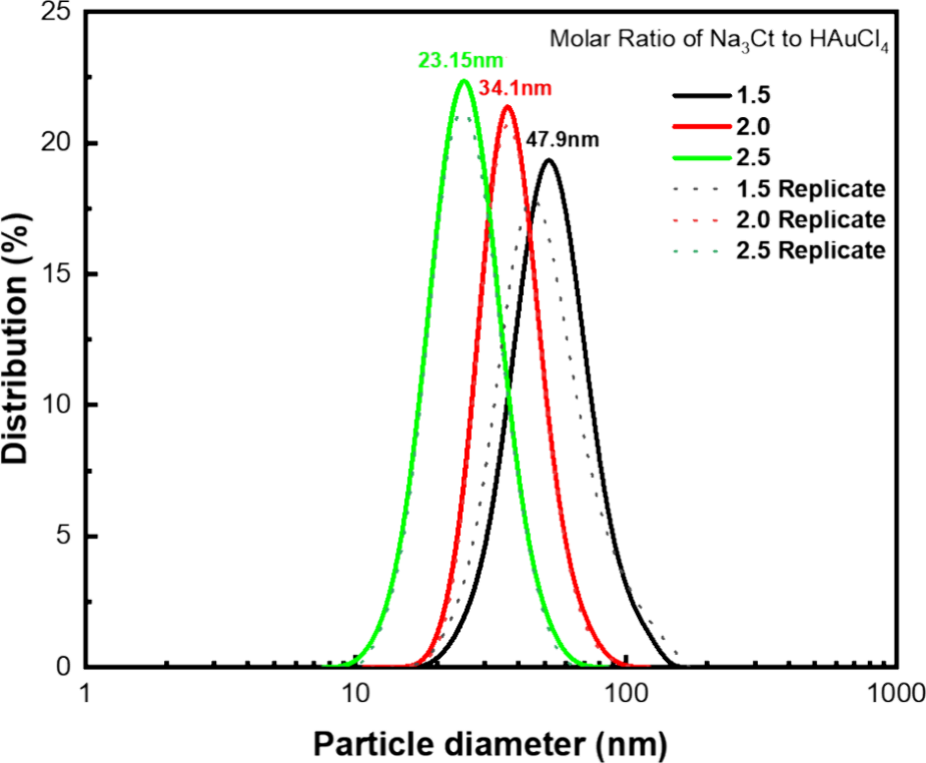
Dynamic light scattering (DLS) measurements show the hydrodynamic size and size distribution of gold nanoparticles with varied molar ratios of Na_3_Ct to HAuCl_4_ (the dashed line represents replicates)_._ The increase in molar ratio resulted in a decrease in hydrodynamic size and narrower size distribution.

In this work, spherical AuNPs were synthesized with varying Na_3_Ct to HAuCl_4_ molar ratios: 1.5, 2.0, and 2.5, resulting in different average particle diameters: 39.6 ± 4.9, 24.7 ± 3.9, and 15.8 ± 2.3 nm respectively, as depicted in the TEM images in Figure 1. It is evident that as the Na_3_Ct to HAuCl_4_ MR increases, the average particle size of the AuNPs decreases. Additionally, DLS measurements in Figure 2 exhibited relatively uniform AuNPs (PDI < 0.15) with narrow distributions and a decreased hydrodynamic size with increasing MR. The control over AuNP sizes by varying the MR may be attributed to the rapid formation of numerous seed particles as the supersaturation of gold atoms increases.^34,36,37^ If the solution contained sufficient citrate, the seed particles would be effectively stabilized, ensuring uniformity in the growth process of AuNPs and their resultant particle size, irrespective of the molar excess. Conversely, as the molar ratio (MR) decreases, the citrate availability for stabilizing the seed particles diminishes. This leads to the aggregation of seed particles, resulting in fewer particles, larger final particle sizes, and less spherical particle morphology. The aggregation process ceases once the particle concentration is significantly reduced. Seed aggregates may induce a self-catalytic effect, facilitating the reduction of Au^3+^ ions on the aggregate surface. Ultimately, growth halts when all precursor material is consumed in the reaction.^34,38^

**Figure 3:**
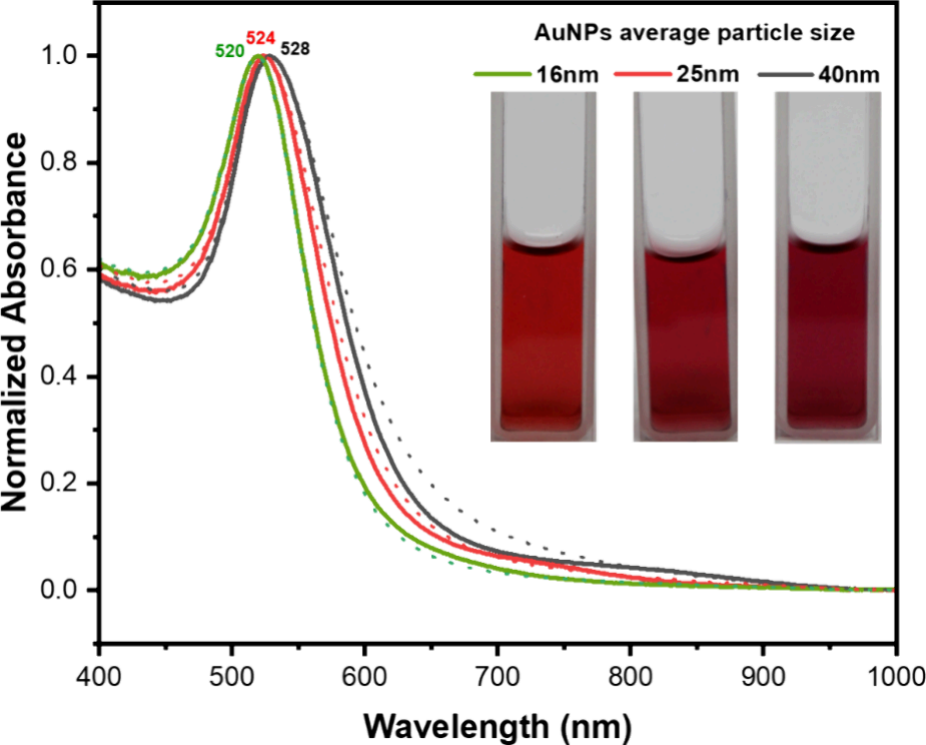
UV–vis absorbance spectra show the surface plasmon resonance peaks of gold nanoparticles of varied sizes (the dashed line represents replicates). The inset displays actual photographs of gold nanoparticles with varied average particle sizes. The increase in the particle size red-shifted the surface plasmon resonance peaks of gold nanoparticles.

The successful formation of AuNPs was indicated by the apparent color change of the precursor solution from yellow to wine-red and the distinctive emergence of a strong surface plasmon resonance (SPR) peak at 520 – 528 nm in the UV–vis spectra, as illustrated in Figure 3. The absorption profile observed is attributed to the characteristic SPR of monodispersed, spherical AuNPs.^39−41^ Furthermore, a shift in the position of the SPR peak toward longer wavelengths, known as a redshift, is apparent with increasing AuNPs size. This might be due to a combination of quantum size effects, alterations in electromagnetic coupling, geometric effects, and plasmon damping.^42,43^ These factors collectively impact the resonance frequency of plasmon oscillations within the nanoparticles.^16^ Consequently, this phenomenon influences our perception of color, resulting in subtle differences in the solution’s hue, as depicted in the inset of Figure 3.

**Scheme 1:**
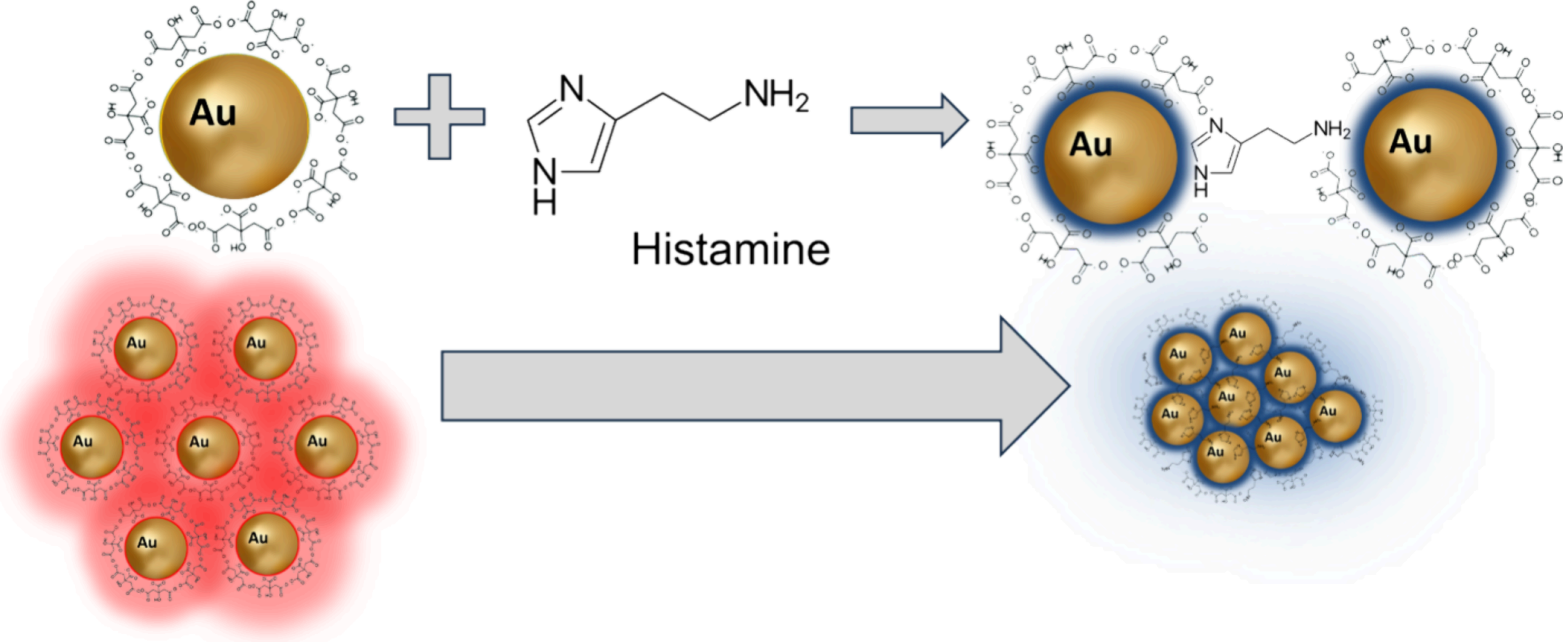
Colorimetric Detection of Histamine Using Citrate-Capped Gold Nanoparticles as a Probe

Standard histamine testing was conducted to validate the feasibility of the synthesized AuNPs sensor for rapid colorimetric detection. Scheme 1 illustrates the mechanism for the colorimetric detection of histamine. The as-prepared citrate-capped AuNPs are stable in an aqueous solution primarily due to the electrostatic repulsion exerted by the negatively charged capping agent, which counteracts the van der Waals attraction between AuNPs. Notably, histamine’s imidazole ring replaces citrate ions due to its strong attraction to AuNPs.^44^ When positively charged histamines bind to the AuNPs, negatively charged citrate ions are released, causing a reduction in the net surface charge of the AuNPs. This can destabilize the AuNPs and trigger aggregation.^29^ The AuNPs solution exhibits a particular color due to the collective oscillations of the surface electrons, which is highly dependent on the interparticle distance.^45,46^ As a result, a color change can be observed when histamine interacts with the AuNP sensor.

**Figure 4:**
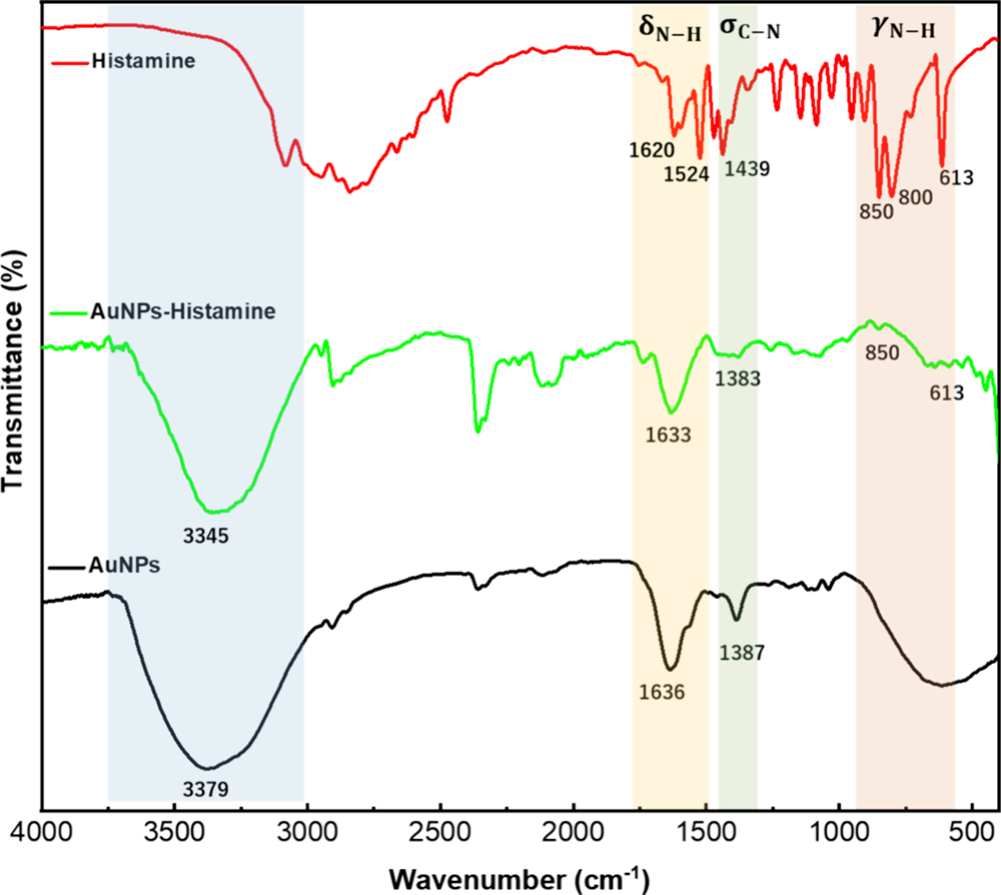
FTIR spectra of AuNPs solution, histamine, and AuNPs with histamine show the OH stretching at ∼3400 cm^–1^, C=O stretching at ∼1630 cm^–1^, and C–O stretching vibrations at ∼1380 cm^–1^, the C=N stretching of amine groups at ∼1600 cm^–1^, the amine bending peak at ∼1520 cm^–1^, the C–N stretching at ∼1430 cm^–1^, and the primary amine peaks between ∼850 and ∼600 cm^–1^, attributed to N–H bending vibrations which become apparent upon AuNPs-histamine interaction.

FTIR was employed to elucidate the mechanism of the interaction between histamine and AuNPs. Analysis of the FTIR spectrum revealed changes in specific functional groups, providing insights into the binding sites and potential reaction pathways involved in histamine detection. As shown in Figure 4, AuNPs have absorption peaks at 1636 and 1387 cm^–1^, which can be attributed to C=O and C–O stretching vibrations, respectively. These peaks are associated with the decarboxylation molecules present in Na_3_Ct that prepare the AuNPs. Histamine exhibits distinctive features in its FTIR spectrum, aiding in its identification. These features encompass the C=N stretching vibration, typically observed in the range of 1650–1600 cm^–1^ (in this instance, observed at 1620 cm^–1^); the amine bending peak at 1524 cm^–1^, arising from the in-plane bending motion of the amine group; the C–N stretching mode, represented by the peak at 1439 cm^–1^; and the primary amine peaks between 850 and 600 cm^–1^, attributed to N–H bending vibrations.

**Figure 5:**
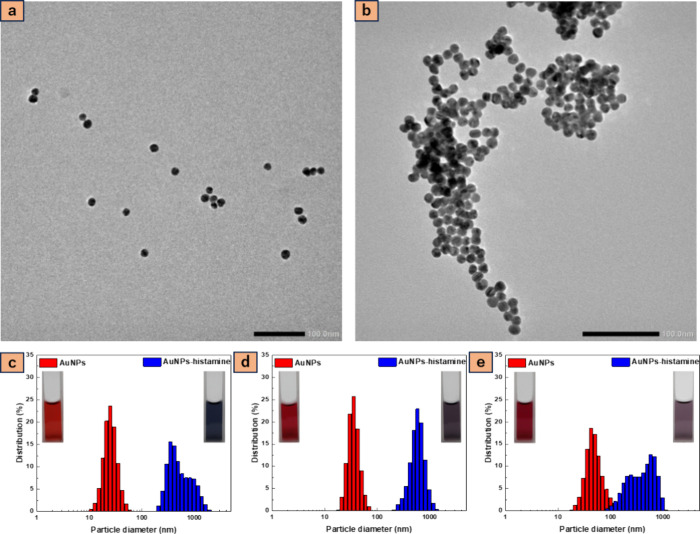
TEM images of (a) gold nanoparticles and (b) aggregation of gold nanoparticles after adding 100 ppm of histamine. DLS measurement of gold nanoparticles solution of varied sizes: (c) 16 nm AuNPs, (d) 25 nm AuNPs, and (e) 40 nm AuNPs with inset images of the colloidal solution. Aggregation of gold nanoparticles is apparent upon the addition of 100 ppm histamine.

Upon interaction with AuNPs, alterations become apparent in the FTIR spectrum of AuNPs-histamine, including the emergence of peaks at 850 and 613 cm^–1^, indicative of γ_N–H_. This suggests the occurrence of interactions between histamine and AuNPs. Subsequently, this interaction induced the aggregation of AuNPs, as evidenced by the morphology depicted in the TEM image shown in Figure 5a,b. Additionally, the DLS measurements illustrated in Figure 5c reveal an augmentation in the hydrodynamic size from 23.15 to 427 nm, an increase in PDI from 0.0795 to 0.2836, and a shift in the zeta potential from −48.7 to +8.8 following the reaction of AuNPs with histamine. Similarly, as shown in Figure 5d, the hydrodynamic size increases from 34.7 to 529 nm, accompanied by an increase in PDI from 0.0938 to 0.1563 and a shift in the zeta potential from −49.2 to +8.8. Likewise, in Figure 5e, the hydrodynamic size increases from 47.9 to 491 nm, with an increase in PDI from 0.1515 to 0.3170 and a shift in the zeta potential from −132.9 to +21.7. This shift in the zeta potential from negative to positive can be attributed to the NH_2_ functional group in histamine, facilitating its attachment to the negative charge of AuNPs, thereby reducing dispersity and promoting aggregation. Consequently, the color of the AuNPs solution changes from red to blue or purple, depending on the size of the AuNPs, as seen in Figure 5 inset images.

**Figure 6:**
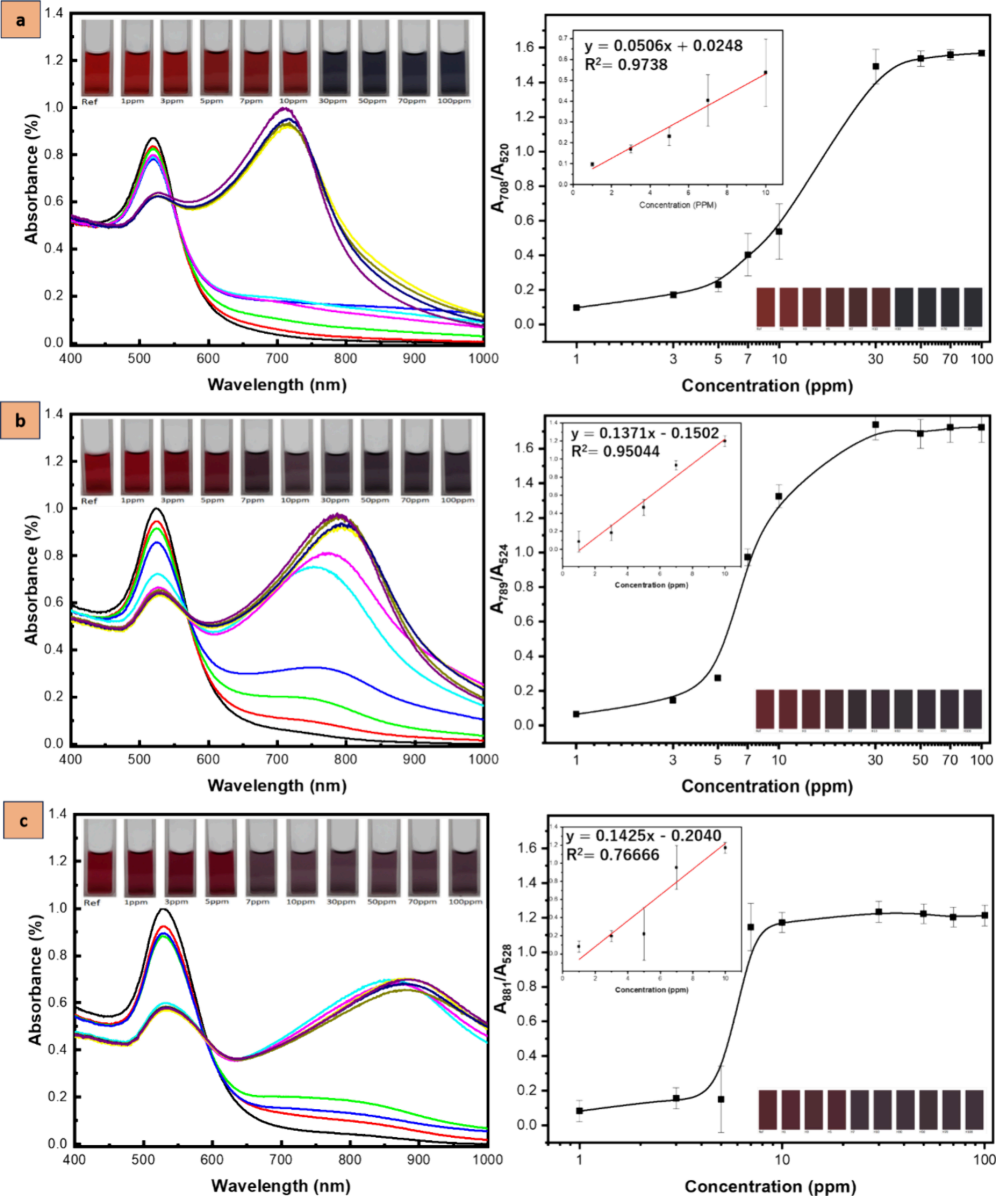
(Left) UV–vis absorbance spectra of (black dashed line) AuNPs solutions of varied sizes: (a) 16 nm AuNPs, (b) 25 nm AuNPs, (c) 40 nm AuNPs after the addition of various histamine concentrations of (red dashed line) 1 ppm, (light green dashed line) 3, (blue dashed line) 5 ppm, (turquoise dashed line) 7, (purple dashed line) 10 ppm, (yellow dashed line) 30 ppm, (olive dashed line) 50 ppm, (indigo dashed line) 70 ppm, and (dark purple dashed line) 100 ppm: (Right) Corresponding absorption ratios of AuNPs solutions. The inset displays the actual solution and its chroma images.

To assess the influence of the AuNP sizes on the colorimetric detection of histamine, various sizes of AuNPs, ∼16, ∼25, and ∼40 nm, were utilized to test different concentrations of histamine ranging from 1 to 100 ppm. Alterations in the absorbance spectra and color changes of the solution induced by the interaction between histamine and AuNPs were observed in Figure 6. It can be monitored from the absorbance spectra that the SPR peak gradually shifts toward a longer wavelength as the concentration of histamine is increased. This is due to the histamine-induced aggregation of AuNPs forming massive networks affecting the interaction of free electrons and photons, greatly enhancing the absorption coefficient in orders of magnitude and changing the SPR absorption.^47^ As a result, the naked eye can observe noticeable color changes from red to blue.

The observed color change of AuNPs is influenced by their size. For instance, the 16 nm AuNPs display a color shift from red to blue (inset of Figure 6a), while the 25 nm AuNPs transition from red to purple-blue (inset of Figure 6b), and the 40 nm AuNPs (inset of Figure 6c) shift from red to purple. This is due to the size-dependent absorption of light by the nanoparticles and the degree of aggregation of each size variation.^32,33^ The nonradiative absorption dominates at small sizes and is caused by the collision of electrons with the nanoparticle surface.^48^ In contrast, the radiative scattering dominates at large sizes, where the damping rate increases with increasing nanoparticle size.^49^ The smaller nanoparticles tend to absorb higher-energy light than the larger ones. Combining the effects of both the absorption and scattering of light results in the observed color changes.^40,50^

To see the details on the impact of AuNPs sizes on the colorimetric detection of histamine, a summary of the diverse sizes of AuNPs obtained through modifications in the Na_3_Ct to HAuCl_4_ molar ratio is presented in Table 1. The table provides information on the respective surface SPR peak wavelengths, detection limits, and histamine concentration observable color shifts, which can be discerned by the naked eye. Among the sizes, 25 nm AuNPs with 524 nm SPR peak have the lowest detection limit of 0.72 μM calculated at a linear range of 1 ppm to 10 ppm with linearity of 0.950. Color response for this sensor is visible at the histamine concentration of 7 ppm with a fast response time of <1 min. This data provides insight into the overall effect of AuNPs sizes on the colorimetric detection of histamine.

**Table 1:** Size, Surface Plasmon Resonance (SPR) Peak Wavelength, Visibly Detectable Histamine Concentrations, and Limit of Detection of Gold Nanoparticles Reduced with Different Na_3_Ct to HAuCl_4_ Molar Ratios (MR)

MR	AuNPs Size		SPR peak wavelength (nm)	histamine concentration inducing distinct color changes (ppm)	limit of detection (μM)
	average particle diameter (nm)	hydrodynamic particle diameter (nm)			
2.5	15.8 ± 2.3	23.2	520	30	1.58
2.0	24.7 ± 3.9	34.1	524	7	0.72
1.5	39.6 ± 4.9	47.9	528	7	1.40

**Figure 7:**
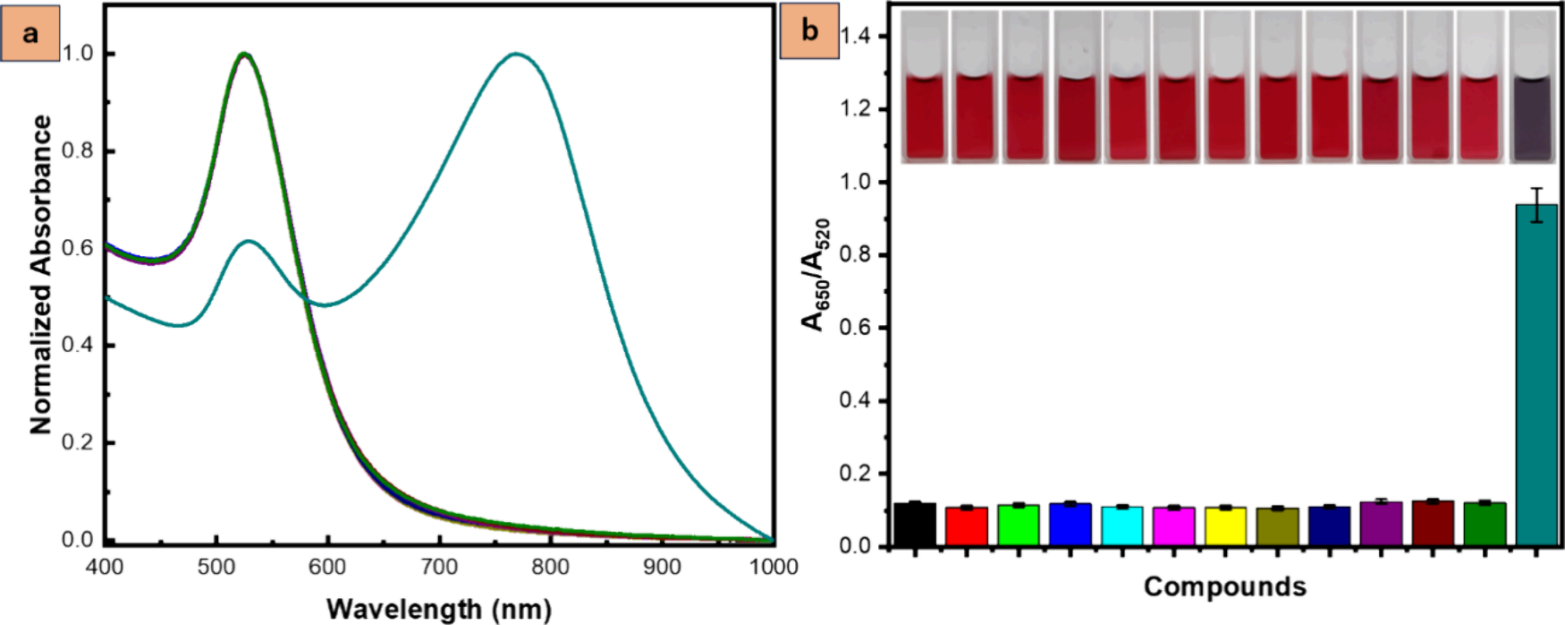
UV–vis spectra show the (a) absorbance and (b) absorbance ratio A_650_/A_520_ of the (black dashed line) 25 nm AuNPs sensor tested with various analytes at 100 ppm such as (red dashed line) CdCl_2_, (light green dashed line) CuSO_4_, (blue dashed line) HgCl_2_, (turquoise dashed line) ZnAc, (purple dashed line) ethanol, (yellow dashed line) methanol, (olive dashed line) ammonia, (indigo dashed line) uric acid, (dark purple dashed line) inosine, (dark green dashed line) cadaverine, and (teal dashed line) histamine, for selectivity assessment.

Investigating the sensor’s selectivity, the 25 nm AuNPs sensor was exposed to diverse analytes relevant to food spoilage (organic solvents, weak acids, other biogenic amines) and other inorganic compounds in our laboratory. Figure 7 shows the UV–vis spectra of these interactions, including exposure to cadmium chloride (CdCl_2_), copper sulfate (CuSO_4_), mercury chloride (HgCl_2_), zinc acetate (ZnAc), ethanol, methanol, ammonia, uric acid, inosine, putrescine, cadaverine, and histamine. UV–vis characterization revealed no significant changes in the AuNP sensor’s absorbance spectra upon exposure to 100 ppm of various analytes, except for histamine. This suggests higher concentrations of other analytes are needed for spectral shifts, highlighting the sensor’s selectivity toward histamine.

**Figure 8:**
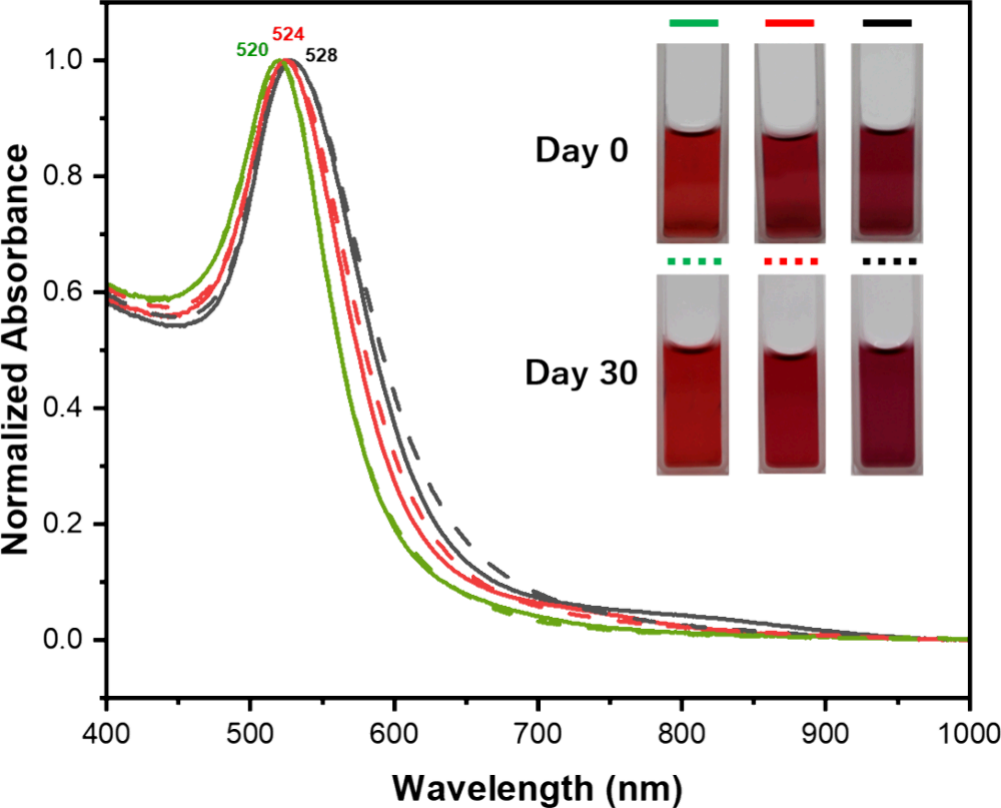
Stability test showing the absorbance spectra of (light green dashed line) 16 nm AuNPs, (red dashed line) 25 nm AuNPs, and (black dashed line) 40 nm AuNPs solution stored at 4 °C after 30 days (dashed lines). The inset displays the actual appearance of the AuNPs solutions. The surface plasmon resonance peak wavelength (520, 524, and 528 nm) remained constant for AuNPs of varied sizes (16, 25, and 40 nm) after 30 days, indicating their stability for extended storage durations.

To assess the colloidal stability of the AuNPs sensor, a time-dependent stability test was conducted by monitoring changes in solution color and the absorbance spectra over one month. Figure 8 illustrates the UV–vis spectra of AuNPs stored at 4 °C for 30 days. The graph indicates no significant alterations in the surface plasmon resonance (SPR) peak of AuNPs after 30 days, signifying that the AuNPs remained well-dispersed. Furthermore, the color of the AuNPs solutions persisted as wine-red and was unchanged throughout this duration. Therefore, the AuNP sensor exhibited sufficient stability for extended storage periods.

Finally, the performance of the AuNP colorimetric sensor was comprehensively assessed by comparing it with existing methods across various parameters including AuNP sizes, linear dynamic range, sensing time, and limit of detection (LOD) values for various biogenic amines. Previously, Bi et al.^23^ demonstrated the detection of histamine using 10 nm-sized AuNPs, achieving a linear range of 0.001–10.0 μM histamine concentration with a detection limit of 0.87 nM after a reaction time of 10 min. Abbasi-Moayed et al.^26^ synthesized 13 nm unmodified AuNPs via the Turkevich method, enabling multiplex detection of spermine, spermidine, histamine, and tryptamine within a linear concentration range of 0.1–10.0 μM, with a histamine detection limit of approximately 0.2 μM after 10 min of reaction time. Lapenna et al.^27^ reported the synthesis of 18 nm “naked” AuNPs via laser ablation in liquid for histamine detection, achieving a linear range of 0.2–0.4 μM histamine concentration with a detection limit of 0.2 μM after 10 min of reaction time. El-Nour et al.^28^ utilized 20 nm citrate-reduced AuNPs to directly detect histamine, achieving a linear range of 0.6–18 μM with a detection limit of 0.6 μM and a sensing time of 15 min.

Accordingly, in this work, the varied sizes of AuNPs were explored as colorimetric sensors for histamine and reported that 25 nm-sized AuNPs exhibit the lowest LOD of 0.72 μM with a linear relationship at a histamine concentration range of 1–10 ppm, having a good correlation coefficient of 0.95 (refer to the inset of Figure 6b). Remarkably, our colorimetric sensor presents a straightforward procedure with rapid response, heightened sensitivity, and exceptional selectivity for histamine detection, achieving a sensing time frame of less than 1 min. This performance surpasses that of most previously reported methods, as detailed in Table 2.

**Table 2:** Comparison of Analyte Responses, Linear Dynamic Range, Sensing Time, and Limit of Detection Value for the Colorimetric Detection of Various Biogenic Amines between Different Literature Studies

sensor probe	sizes	analyte detected	concentration	linear range	sensing time (min)	LOD	references
citrate-capped AuNPs	10 nm	histamine	0.001–10.0 μM	0.001–10.0 μM	10	0.87 nM	(23)
unmodified citrate-reduced AuNPs	13 nm	spermine, spermidine, tryptamine histamine	0.1–10.0 μM	0.1–10.0 μM	10	0.3 μM	(26)
naked AuNPs	18 nm	histamine	0–1 μM	0.2–0.4 μM	10	0.2 μM	(27)
bare citrate-reduced AuNPs	20 nm	histamine	0.6–18 μM	0.6–18 μM	15	0.6 μM	(28)
citrate-reduced AuNPs	16 nm	histamine	0.22–22 μM	0.22–2.2 μM	<1	1.58 μM	this work
	25 nm					0.72 μM	
	40 nm					1.40 μM	

## Conclusions

This study successfully synthesized gold nanoparticles (AuNPs) in various sizes via the citrate reduction method and utilized them as colorimetric sensors for histamine detection. Spherical AuNPs with approximate diameters of 16, 25, and 40 nm, exhibiting strong surface plasmon resonance (SPR) peaks at wavelengths of 520, 524, and 528 nm, respectively, were obtained by adjusting the molar ratio (MR) of Na_3_Ct to HAuCl_4_. However, it is worth noting that this method is limited to synthesizing spherical AuNPs < 50 nm in size, as larger particles tend to lose their spherical shape, exhibit broader size distribution, and produce less reproducible results. Therefore, exploring alternative methods for synthesizing larger AuNPs (>50 nm) is recommended.

Interestingly, the size variations of the AuNPs were found to influence the colorimetric detection of histamine. Notably, the 25 nm-sized AuNPs demonstrated optimal sensor performance, featuring a low detection limit of 0.72 μM within a linear range of 1–10 ppm. This developed colorimetric sensor exhibited rapid sensing capabilities of less than 1 min, alongside excellent sensitivity, stability, and selectivity toward histamine detection. Moreover, by elucidating the effects of different sizes of gold nanoparticles on colorimetric sensing, this study provides insights into optimizing the fabrication of AuNPs-based colorimetric sensors. Additionally, it offers valuable information to researchers aiming to enhance the performance and effectiveness of other colorimetric sensor systems.
